# Well-regulated Dermal Regeneration Using Amnion-containing Scaffold in a Preclinical Study

**DOI:** 10.34172/apb.43476

**Published:** 2025-03-08

**Authors:** Masumeh Staji, Arezoo Karamivandishi, Roya Fattahi, Masoud Soleimani, Hamidreza Moosavian, Simzar Hosseinzadeh

**Affiliations:** ^1^Department of Tissue Engineering and Applied Cell Sciences, School of Advanced Technologies in Medicine, Shahid Beheshti University of Medical Sciences, Tehran, Iran.; ^2^Department of Tissue Engineering and Applied Cell Sciences, Faculty of Advanced Technologies in Medicine, Mazandaran University of Medical Sciences, Sari, Iran.; ^3^Medical Nanotechnology and Tissue Engineering Research Center, Shahid Beheshti University of Medical Sciences, Tehran, Iran.; ^4^Department of Clinical Pathology, Faculty of Veterinary Medicine, University of Tehran, Tehran, Iran.

**Keywords:** Skin tissue engineering, Amniotic membrane, Gelatin, Keratinocytes, Wound healing

## Abstract

**Purpose:**

we investigated the synergistic influence of amnion and keratinocytes on dermal regeneration in mice.

**Methods:**

A scaffold derived from amnion and gelatin via electrospinning was used to synthesize a polyurethane-based scaffold. polyurethane/gelatin/amnion (PU/G/A) scaffold was characterized by scanning electron microscopy (SEM), FTIR, and tensile test. biocompatibility of the corresponding scaffold was investigated using the MTT method in the culture of keratinocytes.

**Results:**

The SEM images showed sufficient cell adhesion on the PU/G/A scaffold. The tensile test results indicated that the scaffold containing PU/G/A with the lowest Young’s modulus (12 MPa±2.1) displayed higher elasticity than the scaffold without amnion. Furthermore, the MTT assay revealed that the amniotic scaffold contributed to 100% cell viability (*P*≤0.0001 compared to control) and proliferation. Moreover, an in vivo study conducted on mice showed that the PU/G/A/ keratinocytes scaffold results in increased granulation, tissue formation and wound closure (*P*<0.001 compared to control).

**Conclusion:**

This innovative nanofiber device not only addresses the limitations of traditional dressings but also offers additional functionalities such as wound compatibility, gas exchange, promotion of angiogenesis in the injured area and a substrate that amplifies the biological functions of stem cells.

## Introduction

 The natural process of wound healing is crucial for restoring the normal function and structure of injured skin. This fundamental physiological process involves the collaboration of different cells, mediators, and growth factors. In cases of minor and uncomplicated wounds the body᾽s innate healing mechanisms are usually capable of fully repairing the damaged area without the need for medical intervention^1^; however, if a large part of the skin tissue is removed due to physical violence, burns, etc.,. The healing process can not heal the resulting ulcer. Therefore, medical care for this type of wound speeds up healing and prevents post-traumatic complications such as infection and water loss.^2,3^ Nowadays, skin tissue engineering is looking for the best skin substitutes using various natural and synthetic materials to promote wound healing in full-thickness burns and non-healing wounds. A high-quality wound dressing possesses distinctive properties that closely mimic the structure of skin and offer sufficient support for cell attachment at the damaged site.

 In addition, the corresponding scaffold must have high biocompatibility, corresponding biomechanical properties, and antibacterial effects. Numerous natural and synthetic biomaterials have been harnessed for the development of wound dressings, each with advantages and disadvantages.^4^ For example, natural biomaterials show high biocompatibility, but when a desired scaffold with unique characteristics is required, these materials are difficult to use due to their low mechanical properties. On the contrary, synthetic materials show higher flexibility and insufficient biocompatibility than natural ones. Blending natural and artificial materials in a scaffold can yield highly effective skin substitutes.^5^

 The amniotic membrane is the innermost layer of the placenta that has been used as a natural scaffold in treating various types of skin wounds for over a century. This biological scaffold has unique properties, making it an excellent injury dressing with the highest similarity to natural skin.^6^ The membrane consists of five layers, including the epithelial layer, basement membrane, compact, fibroblast, and spongy layers. Cells, extracellular proteins, different growth factors and cytokines are present in these layers. positive effect of the amniotic membrane on the wound-healing process is well documented in the literature.^7^ Nonetheless, the absence of adequate mechanical characteristics has presented difficulties in properly protecting the wound site with the amniotic membrane, suggesting using this membrane in combination with different synthetic and natural materials to develop a wound dressing with the desired mechanical behaviors.^8^ A variety of dermal alternatives are presently employed in clinical practice, each with distinct advantages and disadvantages. AlloDerm®, which is a human-derived skin graft obtained from cadavers, offers natural skin porosity and preserves the basal membrane, enabling host skin cells to migrate and adhere effectively. However, it poses risks such as the potential for infectious disease transmission, the requirement for multiple surgeries, and elevated costs. In contrast, Integra® is a synthetic skin transplant made from bovine collagen and chondroitin-6-sulfate, providing a dermal scaffold that mimics human skin. This allows the host to create its own skin layer, leading to better aesthetic and functional results. Nonetheless, Integra has its own challenges, including the lack of an epidermal component, the risk of fluid buildup and subsequent infections, and again, high costs. Despite progress in this area, an ideal skin alternative remains elusive.^9^

 Gelatin is produced by partial hydrolysis of animal collagens from different tissue sources such as bone, tendon, and skin.^8^ Based on the hydrolysis approaches, there are two types of gelatins, including type A and B, deriving from acid and alkaline hydrolysis of collagen, respectively. Gelatin has become a popular natural biomaterial in tissue engineering (TE) because of the remarkable diversity in the physical properties of this material, which can be attributed to differences in collagen sources and production methods. In addition, gelatin shows high biocompatibility and biodegradability, low antigenicity, and an increased number of adhesion motifs and functional groups, making this material accessible to blend with various synthetic and natural materials.^10^

 Polyurethane (PU) is one of the most prominent families of polymers, which can be obtained from a wide variety of sources and, therefore, have different physico-chemical properties. These polymers are widely used in various biomedical fields, from cardiac pacemakers to scaffolds for tissue engineering.^11^ PU-based wound dressings have recently gained an acceptable market position due to their remarkable properties, such as gas permeability and avoidance of water loss at the wound surface. Combining PU with different synthetic and natural materials has been associated with notable outcomes in skin engineering.^12^

 The current research involved the fabrication of a state-of-the-art nanofibrous matrix using electrospinning technique, comprising PU, gelatin, and amnion. To measure the cellular behavior of electrospun polyurethane/gelatin/amnion (PU/G/A) nanofibers, the cell attachment of keratinocytes in the nanofiber matrix and the interaction between cells and PU/G/A nanofibers were investigated. Additionally, the role of PU/G/A nanofibers in the healing process of open wounds in mice was explored.

## Material and Methods

###  Preparation of polymer

 The homogeneous solution of aliphatic polyether-based thermoplastic polyurethanes (TPU) at a concentration of 10% w/v was prepared by dissolving Tecoflex^®^ SG-80A (Lubrizol, USA) in a mixture of Tetrahydrofuran (THF) and Dimethylformamide (DMF) (70:30) and stirring overnight. Similarly, gelatin solution was prepared at a concentration of 20 wt.% by adding a calculated amount of Gelatin (Sigma-Aldrich, USA) in 40% acetic acid (Merck, USA) and stirring for 1 hour. The amnion solution (5%, w/v) was obtained by dissolving a calculated amount of amnion micronized powder (by freeze-drying the amniotic membrane) in distilled water and sonicating it for 20 min. This amnion solution was then mixed with the gelatin solution.

###  Process of electrospinning

 The electrospinning process was carried out to prepare two distinct electrospun composite meshes, including (a) Co-electrospun gelatin and TPU and (b) Co-electrospun gelatin/amnion/PU as following conditions: The flow rate was 0.2 mL/h while there was 15 cm air gap between the rotating mandrel and the 18-gauge needle tip. 15 kV of high-voltage power and rotational speed of 300 RPM was supplied. After preparation of the developed meshes, they were air dried at room temperature overnight to remove the residual DMF. Later, electrospun meshes were exposed to glutaraldehyde vapor (10 mL of 1% glutaraldehyde) to cross-link the gelatin fibers. The function of this particular cross-linking was examined by immersing the samples in phosphate-buffered saline (PBS). The cross-linked nanofibers were rinsed with Deuterium-Depleted Water (DDW) three times to remove residual chemicals.

###  Scaffold morphology

 The morphological characteristics of PU/G/A and polyurethane/gelatin (PU/G) scaffolds were evaluated using scanning electron microscopy (SEM) (Philips XL30; Philips, Eindhoven, Netherlands). The nanofibers’ average diameter and pore size were computed.

###  Contact angle test

 The contact angle between water drops and electrospun PU/G and PU/G/A membranes was evaluated by the VCA Optima contact angle system mounted with a video cam.

###  Tensile test

 The mechanical properties and recovery of the blended PU/G nanofiber scaffold were estimated using tensile and healing by an Instron machine (Instron 5566, Canton, MA, USA) and a device fabricated by our laboratories, respectively. The laboratory assessed the mechanical characteristics of cross-linked and uncross-linked PU/G and PU/G/A scaffolds. dried specimens were cut into approximately 20 mm × 5 mm × 0.5 mm (length × width × thickness to be loaded into the uniaxial testing machine. The samples were measured with a 10 N load cell under a cross-head speed of 10 mm/min(n = 3).

###  Cell culture and preparation

 Human Epithelial Keratinocytes primary cells isolated from the dermis layer of the skin were cultured in Dulbecco’s modified eagle’s medium (DMEM), supplemented with 10% bovine serum and incubated at standard conditions (37 °C and 5% CO2). cultured medium was replaced with fresh medium every two days.

###  Cell viability

 The rounded electrospun scaffolds were placed in 24-well plates and sterilized with 75% alcohol for 2 h. Then, they were washed with PBS and exposed to UV. In the next step, keratinocytes with a density of 10 × 10^3^ cells/cm^2^ were seeded on the developed scaffolds. cell viability after 1, 3, 5, 7, and 14 days was evaluated using an MTT assay.

###  Cell morphology 

 After 1 and 7 days of cell culture, SEM topography was conducted to study cell morphology on PU/G and PU/G/A scaffolds. Briefly, samples were fixed with 2.5% glutaraldehyde overnight at 4 °C. After fixation, the samples were washed three times with distilled water and then dehydrated in an ethanol/distilled water mixture from 50% to 100% each step for 10 minutes. After dehydrating, the samples were lyophilized for 3 days. The obtained samples were observed using a Hitachi SEM (S-2300, Tokyo, Japan).

 PU/G and PU/GA scaffolds containing keratinocytes were fixed with 4% paraformaldehyde for 15 minutes at room temperature after washing with PBS and then stained with 4,6-diamidino-2-phenylindole (DAPI, 1:1000) were stained. (Life Technologies, Carlsbad, CA, USA). The samples were examined using a confocal microscope (Leica Microsystems, Wetzlar, Germany).

###  In vivo study

 This study used two types of PU/G/A and PU/G scaffolds for grafting. Keratinocytes at a concentration of 1 × 10^4^ were cultured on scaffolds measuring 6 mm in diameter for a period of 7 days. The experiment involved the use of 12-14 week old male mice weighing 20 ± 5 grams (n = 15). Mice were housed under identical conditions for two weeks to minimize the negative effect of stress caused by the unfamiliar environment on the study results. Anesthesia was induced with 5 mg/kg xylazine hydrochloride 2% and 5 mg/kg ketamine hydrochloride 10% intraperitoneally. The dorsal surface of the mice was prepared and shaved, and a circular lesion measuring 5 mm in diameter was formed on the posterior aspect of the neck. By employing biopsy punches, the injury was completely excised. Following the injuries, mice were randomized into 5 treatment group: PU/G scaffold with keratinocytes, PU/G/A scaffold with keratinocytes, PU/G scaffold without keratinocytes, PU/G/A scaffold without keratinocytes, and control (Vaseline/gas).

 After creating a wound, separating and removing the skin from the subcutaneous tissue, scaffolds were placed on the wound area and then used sutures and sterile gauze to fix it. Wound healing was evaluated on days 0, 1, 7, and 14. On the 14th day, the animals were euthanized, and the wound area was dissected and made ready for H & E staining.

###  Statistical analysis

 All investigations in this study were repeated three times, and the test and control groups were compared using a t-test. The obtained *P* value of ≤ 0.05 was used to assess the significance of the results. All the reported data are the average of the obtained numbers and are displayed as the mean ± standard error.

## Results and Discussion

###  Scaffold morphology

**Figure 1:**
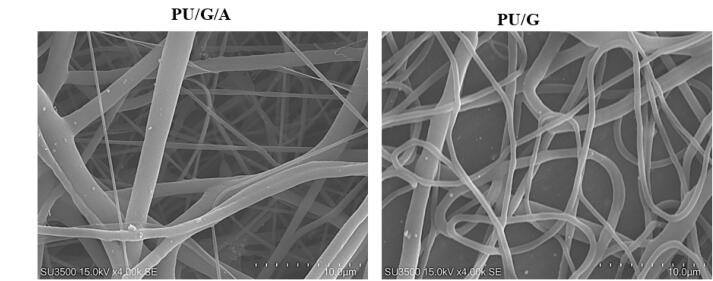
SEM images of PU/G/A and PU/G at a scale of 10 μm

 The morphology of the electrospun scaffolds plays an essential role in cell attachment and migration to the scaffold. In Figure 1, the PU/G/A scaffold showed an average fiber diameter of 245 nm in the 150–390 nm range, and the PU/G scaffold showed an average fiber diameter of 320 nm in the 148–437 nm. In the PU/G/A scaffold, nanofibers showed a smaller average diameter, which is attributed to the amnion’s effect on the electroconductivity of the electrospun solution. On the other hand, this nanostructure is sufficient to facilitate cell attachment. In a similar study, by adding dextran to the matrix, PU decreased the fiber diameter and increased cell adhesion and proliferation.^13^ Furthermore, in previous studies, adding amnion led to forming a homogeneous and nanofibrous structure without negatively affecting the morphology.^14^ All investigations in this study were replicated three times.

###  Contact angle test

**Figure 2:**
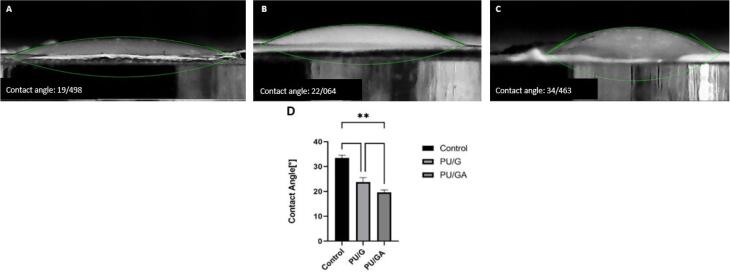
Water contact angle measurements of A: PU/G/A scaffold, B: PU/G scaffold, C: Control group. Statistical significance is indicated as follows: **P < 0.01

 The hydrophilic property of an electrospun scaffold is essential for biomedical use and especially for wound healing.^15^ high hydrophilicity of the designed scaffold was due to the properties of amnion and gelatin. behavior of the scaffold surface towards water droplets is known by the “contact angle” index, which indicates the degree of cell adhesion to the scaffold substrate. active functional groups and the physical properties of the scaffold regulate their amount.^16^ contact angles of the nanofibrous scaffolds without and with amnion were 22.06° and 19.49°, respectively (Figure 2). It was observed that adding amnion to PU and gelatin reduced the contact angle, indicating the wettability properties of these scaffolds compared to the control group (*P* < 0.05), however this difference between the two PU/G and PU/G/A scaffolds was not significant (*P* > 0.05). It is clear that the amnion exhibits a higher level of hydrophilicity.^17,18^ because the amniotic membrane’s extracellular matrix is composed of collagen types I, III, IV, V, and VII, which has led to improved hydrophilic characteristics.^6^

###  Tensile test

**Figure 3:**
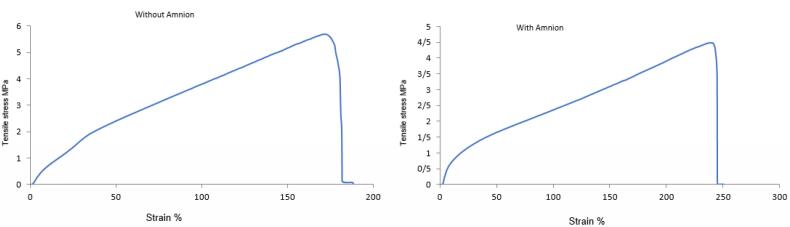
Tensile stress–strain curves for PU/G/A (right) and PU/G (left) scaffolds

Figure 3 indicates the mechanical properties of the PU/G/A and PU/G scaffolds. The low strength of natural nanofiber scaffolds makes them a popular choice for combination with synthetic polymers. The comparative tensile strength diagram showed that the PU/G/A scaffold can withstand a higher percentage elongation (247 ± 9) than the PU/G scaffold (188 ± 12), indicating greater flexibility.^19^ Moreover, one can conclude that the PU/G/A scaffold is designed to handle smaller loads and has a reduced ultimate tensile strength of 4.2 ± 1.8 MPa, whereas the PU/G scaffold shows a higher strength of 5.6 ± 1.1 MPa. The calculated value of Young’s modulus for the PU/G/A scaffold (12 ± 2.1 MPa) and PU/G (48 ± 3.2 MPa) showed that the scaffold with amniotic has higher elasticity compared to the scaffold without amnion while maintaining the integrity of the structure concerning the applied load. Compared with the maximum tensile strength of the human amniotic membrane, the PU/G/A scaffold has good elasticity and tensile strength to resist skin cell transfer. It can be an excellent 3D substrate for the culture of skin cells.^20^ In addition, the simulation of this scaffold type is very similar to the in vivo conditions.^21^ The incorporation of amnion has been shown to induce softening, which is defined by a low modulus of elasticity.^22^

###  Cell attachment

**Figure 4:**
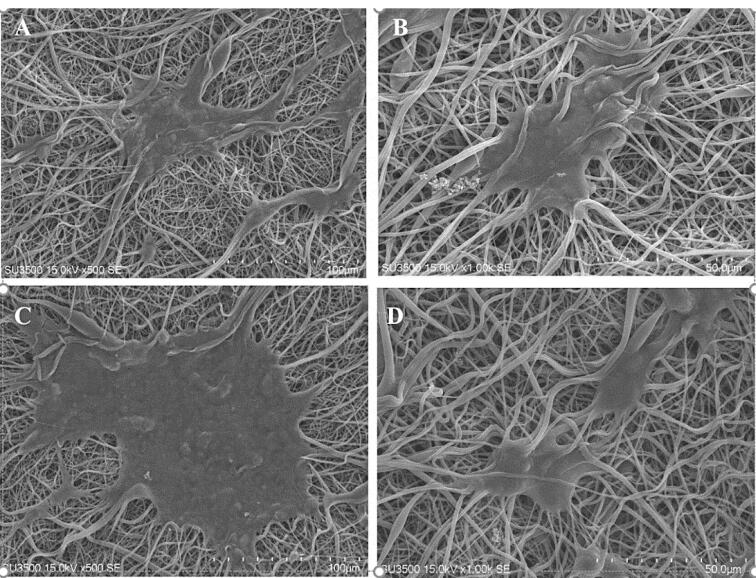
SEM micrographs of seeded cells on PU/G (A, B) and PU/G/A (C, D) scaffolds on day 7

**Figure 5:**
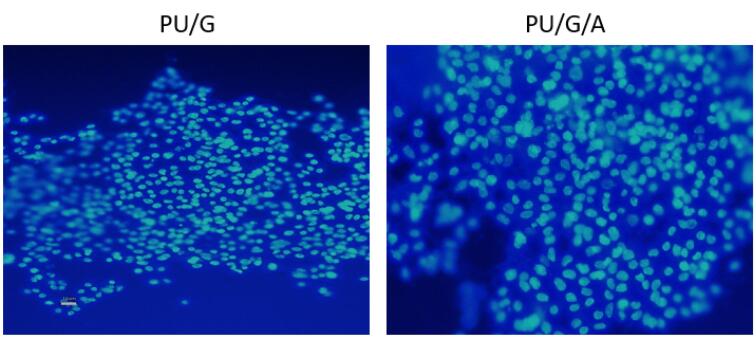
DAPI stained images of keratinocytes cultured on PU/G and PU/G/A scaffold on day 7

 SEM observations evaluated the keratinocyte morphology after seven days of culture on the scaffolds. As seen in Figure 4, the adhesion of the cells was observed on the PU/G/A scaffold with more filopodia than on the PU/G scaffold, and the cells became completely wide and flat and attached to the surface of the scaffold. The scaffold’s superior adhesion can be attributed to the presence of amnion, facilitating better cell attachment. Epithelial or mesenchymal cells implanted on the scaffold created from the amniotic membrane were strongly interconnected and could penetrate the porous structure. They have also been used for vascular regeneration in the skin.^23^ In the DAPI image (Figure 5), the PU/G/A scaffold, compared to PU/G, showed more cell proliferation and adhesion. Owing to the amnion’s presence, the cells had better proliferation and migration. The nanocomposite scaffold, including amnion, increased cell adhesion and proliferation.^24^ The amniotic membrane matrix with extracellular proteins such as collagens, laminins, and fibronectins plays a crucial role in wound healing.^25^ In fact, the combination of a porous architecture and favorable mechanical properties in amnion-containing scaffolds may enhance the release of molecular signals, potentially impacting the absorption and migration of cells toward the substrate.^26^ All investigations in this study were replicated three times.

###  Cell viability

**Figure 6:**
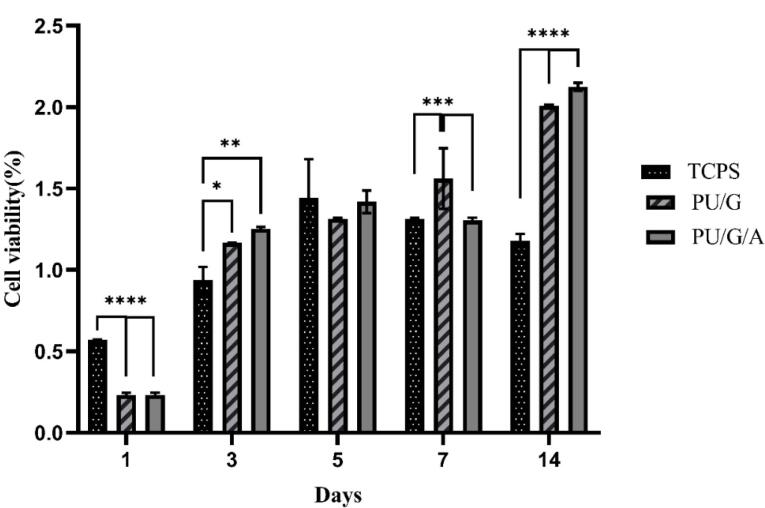
The histogram of MTT assay, comparing the survival of keratinocytes cultured on PU/G, PU/G/A scaffolds and control group (TCPS) on days 1, 3, 5, 7, and 14. Statistical significance is indicated as follows: *P < 0.05, **P < 0.01, ***P < 0.001, ****P < 0.0001

 The MTT method evaluated the adhesion and primary cell proliferation on PU/G and PU/G/A scaffolds. Figure 6 shows the optical intensity of the culture with keratinocytes seeded on both scaffold substrates for 1, 3, 5, 7, and 14 days. The total number of keratinocytes increased after 3,7 and 14 days. After 1 and 3 days, both PU/G and PU/G/A scaffolds were significant compared to tissue culture polystyrene (TCPS). However, the PU/G/A scaffold did not show a substantial difference with PU/G. On 5 days, the scaffolds did not differ significantly from each other and the TCPS. Following a week, the PU/G scaffold exhibited a marked difference from TCPS, unlike PU/G/A. On 14 days, both scaffolds were significantly different from TCPS but not with each other. Cell populations on the PU/G/A scaffold demonstrated more extraordinary proliferation ability than those on PU/G. This observation provides evidence that amnion increased cell viability. cell proliferation rate in the membrane derived from amnion increased faster.^20^ The human amniotic membrane has 100% cell viability due to non-toxicity.^27^ The amnion promotes cell proliferation through cell-matrix interactions.^28,29^

###  In vivo study

**Figure 7:**
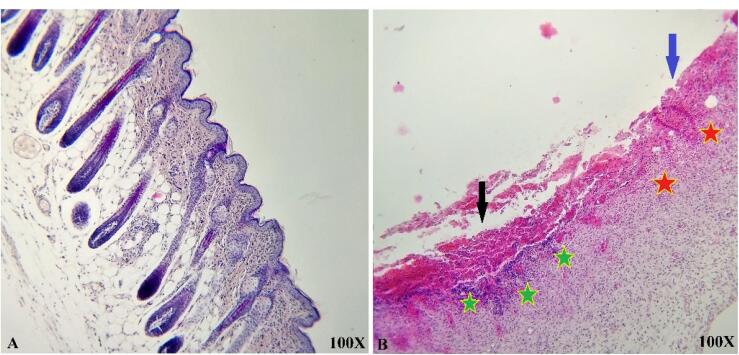
Representative histological images of intact skin (A) and negative control sections from day 7 (B). Hemorrhage (Black arrow), necrosis (Blue arrow), partial granulation tissue formation (Red star), and dermal infiltration of inflammatory cells (Green star) were present

**Figure 8:**
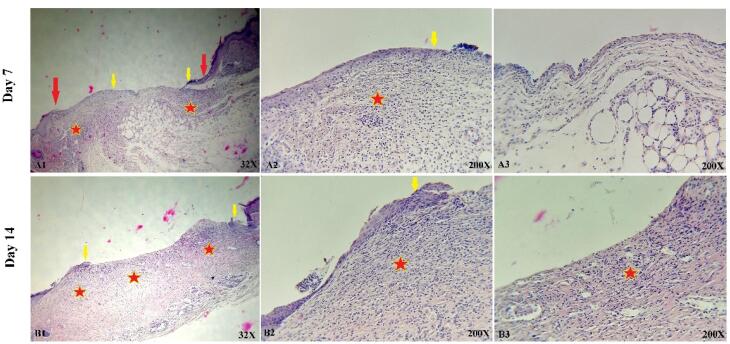
Representative histological illustrations of skin sections from wounds treated with PU/G without cells, captured on days 7 (A1-A3) and 14 (B1-B3). Epithelial skin edges are marked by red arrows, while the lots of re-epithelialization are indicated by yellow arrows. Additionally, granulation tissue formation is represented by red stars

**Figure 9:**
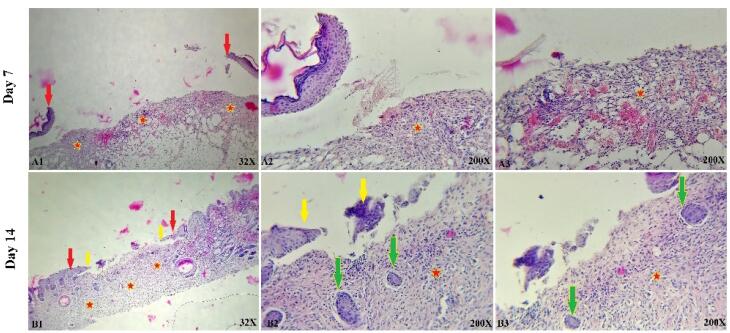
Histological images of skin sections from wounds treated with PU/G containing keratinocytes are presented for days 7 (A1-A3) and 14 (B1-B3). Red arrows denote the edges of epithelial skin, yellow arrows indicate the ends of re-epithelialization, epithelial cell nests in the dermis are represented by green arrows and red stars denote granulation tissue formation

**Figure 10:**
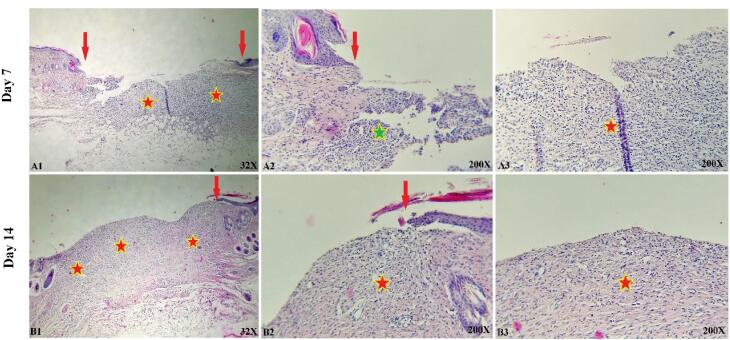
Representative histological images of skin sections from wounds treated with PU/G/A without cell for days 7 (A1-A3) and 14 (B1-B3). Red arrows denote the edges of epithelial skin, red stars denote granulation tissue formation, and green stars indicate dermal infiltration of inflammatory cells

**Figure 11:**
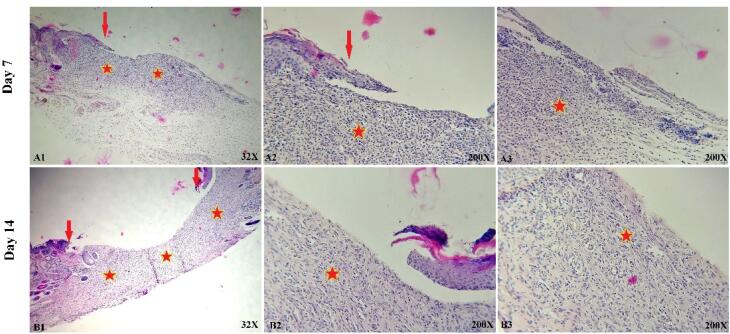
Representative histological images of skin sections from wounds treated with PU/G/A with seeded keratinocyte for days 7 (A1-A3) and 14 (B1-B3). Red arrows denote the edges of epithelial skin; red stars denote granulation tissue formation

 The histological and microscopic evaluation of the samples was done by staining H&E. Figure 7, and the macroscopic images of intact skin (A) and negative control sections (B) were indicated. In the negative control group, bleeding, tissue granulation, necrosis, and secretion of inflammatory cells were observed. No cells were observed in the PU/G treatment group depicted in Figure 8. Throughout a week, the wound exhibited the formation of granulation tissue (red stars) and re-epithelialization (yellow arrow) at the wound edges. Interestingly, no granulation tissue formation was observed in the central area of the wound (A1-A3). On day 14, there was granulation tissue formation throughout the injury, and collagen production was higher compared to day 7 and also was re-epithelialization. In Figure 9, keratinocytes were seen with the PU/G scaffold. On 7 days, high granulation tissue formation at the wound’s edges and low granulation tissue formation at the wound’s center was shown (A1-A3). The injury site demonstrated the presence of granulation tissue formation throughout the 14-day period, with a marked increase in collagen production when compared to day 7 (B1-B3). Figure 10 shows the treatment group with PU/G/A scaffold without cells. During the course of 7 days, there was evidence of partial granulation tissue formation and dermal infiltration of inflammatory cells at the edges of the epithelial skin, as well as granulation tissue formation in the center of the wound. (A1-A3). On 14 days, granulation tissue formed throughout the wound, and collagen production was higher than on day 7. Red arrows denote the ends of epithelial skin (B1-B3). Figure 11 shows the treatment group with PU/G/A scaffold with keratinocytes. On 7 days, partial granulation tissue was formed in the ends and wound center (A1-A3). On 14 days, granulation tissue formed throughout the wound, and collagen production was higher than on day 7 (B1-B3). In vivo, the application of amnion relieves pain, allows faster wound healing and promotes re-epithelialization.^30^ Using amnion in the scaffold reduced immunogenicity and toxicity.^31^ Microvessel densities that were mature and stable and re-epithelialization increased.^32^ A wound is characterized by the absence of tissue continuity. Re-epithelial formation is observed throughout the entire surface of the injury.^33^ The amniotic membrane acts as the basement membrane. Consequently, using an amniotic membrane on the PU/G/A scaffold facilitates the adhesion and differentiation of epithelial cells and ultimately prevents apoptosis.^34^ Inflammation is the starting point of the wound healing process, and it is expected to gradually subside.^35^ During wound healing, early-stage inflammatory cells increased and gradually reduced due to granulation formation of new capillaries and collagen deposition, similar to previous studies.^36^ A meta-analysis of eleven randomized controlled trials with 816 participants revealed that the use of amniotic membrane treatment was more effective than standard methods, silver sulfadiazine, and PU membrane in addressing burn wounds. However, it was found to be less effective than honey. Importantly, none of the articles reviewed reported any disease transmission or adverse reactions associated with amniotic membrane.^37^

 Histological analysis of our experiment showed that the amnion-containing group had a higher rate of epithelialization. Since wound healing is related to wound contraction and regeneration,^38^ amniotic scaffolds have been shown to heal wounds in a mouse mode.^39,40^

###  Wound closure

**Figure 12:**
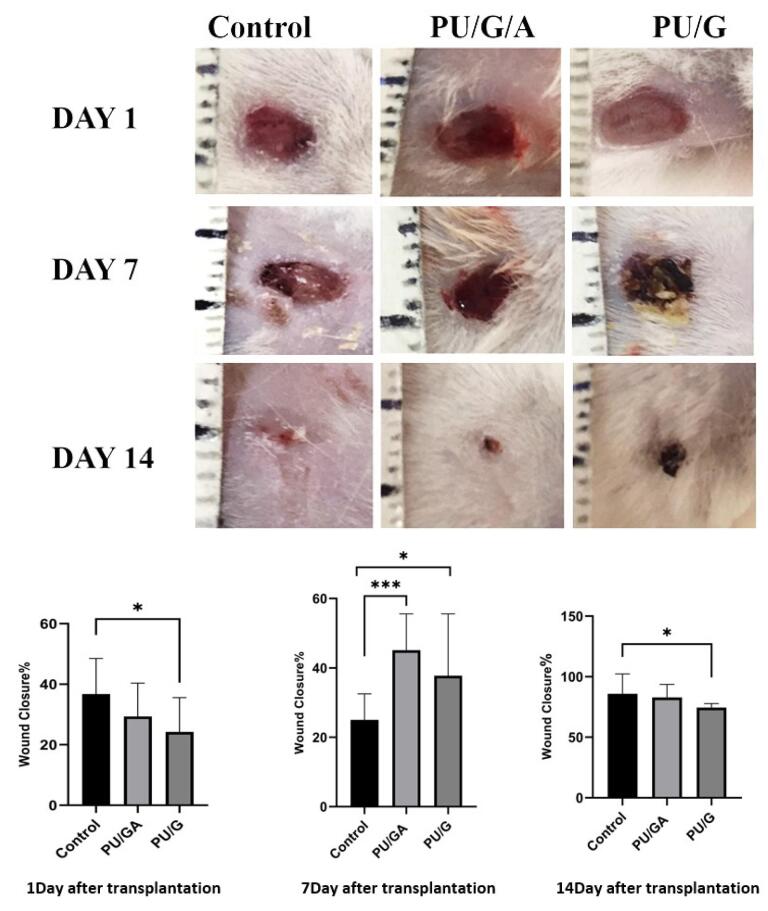
Macroscopic evaluation of wound closure in a day-dependent and treatment-dependent perspectives and statistical analysis of wound closure on days 1, 7, and 14. Statistical significance is indicated as follows: *P < 0.05, ***P < 0.001

 Skin wounds of animals treated with PU/G/A and PU/G scaffolds and keratinocytes (control) were digitally recorded on days 1, 7, and 14 after surgery (Figure 12). Wound closure was evaluated by wound size reduction on day 1, 7, 14 in all groups. The mean wound reduction in the control group on days 1, 7, and 14 was 36.77, 25.02 and 85.96%, respectively, and in the PU/GA scaffold group with keratinocytes on days 1, 7, and 14 were respectively 29.37, 45.10, 82.92% and in the PU/G scaffold group with keratinocytes, on days 1, 7, and 14, it was respectively 24.24, 35.75, and 74.52%. The histological results’ evaluation showed the amnion’s role in wound healing. The PU/G/A/keratinocytes scaffold increased granulation, tissue formation, fibroblast proliferation, production of large numbers of collagen fibers, and vascular sprouting, which are especially necessary for successful tissue regeneration^41^ and prevent healing necrosis.^42^

## Conclusion

 This study showed that amnion on the electrospun nanofiber scaffold increased the proliferation and differentiation of keratinocytes. Amnion also converted the PU/G/A scaffold with high hydrophilicity, providing a suitable substrate for cell adhesion and biocompatibility. It had proper mechanical strength for use in skin repair. The histological results displayed the sequential processes of wound healing, such as cell proliferation, migration, angiogenesis, epithelialization, and tissue regeneration. Collagen fibers are formed during the regeneration phase, making the PU/G/A scaffold and keratinocytes an ideal combination for reducing scarring during wound healing. consequently, the current study sheds light on the utilization of PU/G/A nanofiber technology for the purpose of wound healing treatment.

## Competing Interests

 The authors declare no competing financial and non-financial interests.

## Ethical Approval

 This study was approved by the ethical committee of Shahid Beheshti University of Medical Sciences, Tehran, Iran, with the ethical code of IR.SBMU.RETECH.REC.1397.422.
